# Favourable ten-year overall survival in a Caucasian population with high probability of hereditary breast cancer

**DOI:** 10.1186/1471-2407-10-90

**Published:** 2010-03-10

**Authors:** Laura Cortesi, Cristina Masini, Claudia Cirilli, Veronica Medici, Isabella Marchi, Giovanna Cavazzini, Giuseppe Pasini, Daniela Turchetti, Massimo Federico

**Affiliations:** 1 Department of Oncology and Haematology, University of Modena and Reggio Emilia, Modena, Italy; 2 Modena Cancer Registry, Modena, Italy; 3 Department of Oncology and Haematology, Carlo Poma Hospital, Mantua, Italy; 4 Department of Medical Oncology and Oncohaematology, City Hospital, Rimini, Italy; 5 Medical Genetic Service, University of Bologna, Bologna, Italy

## Abstract

**Background:**

The purpose of our study was to compare differences in the prognosis of breast cancer (BC) patients at high (H) risk or intermediate slightly (IS) increased risk based on family history and those without a family history of BC, and to evaluate whether ten-year overall survival can be considered a good indicator of *BRCA1 *gene mutation.

**Methods:**

We classified 5923 breast cancer patients registered between 1988 and 2006 at the Department of Oncology and Haematology in Modena, Italy, into one of three different risk categories according to Modena criteria. One thousand eleven patients at H and IS increased risk were tested for *BRCA1/2 *mutations. The overall survival (OS) and disease free survival (DFS) were the study end-points.

**Results:**

Eighty *BRCA1 *carriers were identified. A statistically significantly better prognosis was observed for patients belonging to the H risk category with respect to women in the IS and sporadic groups (82% vs.75% vs.73%, respectively; p < 0.0001). Comparing only *BRCA1 *carriers with *BRCA-*negative and sporadic BC (77% vs.77% vs.73%, respectively; p < 0.001) an advantage in OS was seen.

**Conclusions:**

Patients belonging to a population with a high probability of being *BRCA1 *carriers had a better prognosis than those with sporadic BC. Considering these results, women who previously had BC and had survived ten years could be selected for *BRCA1 *analysis among family members at high risk of hereditary BC during genetic counselling. Since only 30% of patients with a high probability of having hereditary BC have *BRCA1 *mutations, selecting women with a long term survival among this population could increase the rate of positive analyses, avoiding the use of expensive tests.

## Background

The major breast cancer (BC) predisposing genes, *BRCA1 *and *BRCA2 *were identified in 1994 and 1995, respectively [1,2]. Unfortunately, the optimal clinical approach to women who develop hereditary breast cancer remains incompletely defined. Studies of the outcomes of women with *BRCA1*/*BRCA2*-related cancer have yielded conflicting results. Several reports suggested that women with germline mutations in *BRCA1 *are more likely to die from their disease than are women with sporadic breast cancer [3-6], whereas *BRCA2 *mutation carriers and non-mutation carriers seem to share a similar prognosis [7,8]. The poor prognosis in *BRCA1 *carriers may be consistent with the histological characteristics usually described for *BRCA1*-associated breast cancer, which show higher histologic grade and cancers that are more often hormone receptor-negative than sporadic breast cancer cases [9-12]. However, Bonadona et al. found no evidence for poorer short-term survival in *BRCA1 *mutation carriers compared with non-carriers in a prospective population-based cohort [13]. Apart from a simple interest in the epidemiological aspects of breast malignancy, knowledge of the associated mortality is important to the families of patients with BC and to clinicians and scientists involved in trying to improve the outcomes of breast cancer. The results of a recent study in an Ashkenazi Jewish population suggested that among the subgroup of patients with BC carrying a *BRCA1 *mutation, those who received chemotherapy had a better survival rate compared with those who did not [8].

The primary aim of our study was to calculate disease free survival (DFS) and overall survival (OS) of BC patients at high risk (H) or intermediate slightly (IS) increased risk based on family history and those without a family history of breast cancer using the population registered with the Breast Cancer Registry in Modena. Previous studies were aimed at evaluating the outcome in *BRCA*-positive and *BRCA-*negativepatients, but none showed a significant survival difference between different risk categories. In case of statistically significant differences in OS between the three groups, a secondary aim was to determine whether patients with a better prognosis were *BRCA1 *mutation carriers, showing that the outcome could be considered an indicator of *BRCA1 *inheritance. Additionally, we evaluated whether chemotherapy could play a role in the prognosis of *BRCA1 *carriers, providing more benefit in this patient population than in patients with sporadic breast cancer.

## Methods

### Patients

Patients included in our analysis were diagnosed between 1988 and 2006 at the Department of Oncology and Haematology in Modena. All newly-diagnosed, biopsy-proven primary breast cancer patients were evaluated. The family history was collected and classified according to the Modena criteria (without family history, IS increased risk, or H risk)[14] and a blood sample, preserved with EDTA, was obtained with informed consent and frozen at -80°C for biological studies. On the basis of the Modena criteria for familial risk, patients who did not have any family history were considered to have sporadic breast cancer, patients with one or two breast cancers at ≥ 40 years, without a first-degree relationship, were considered at IS increased risk, and patients with breast cancer at ≤ 35 years, three or more breast cancers with a first-degree relationship, and at least one case at ≤ 40 years or bilateral breast cancer had a H risk of being hereditary.

All research regarding the identification, counselling, genetic testing, and clinical data regarding individuals at risk of developing breast cancer were ethically approved by the Ethics Commitee of Modena (reference number 45/00).

### Mutational analysis

In 1995, DNA started to be extracted from frozen whole blood using the Invisorb Blood Universal kit (Invitek, Berlin, Germany), amplified by PCR using primers specific for the coding sequence and exon-intron boundaries of *BRCA1*, and analyzed by Direct Automated Sequencing using an ABI Prism 3100 (Applied Biosystems, Foster City, CA). Subsequently, patients with breast cancer provided consent for genetic testing that was completed for each case.

Samples negative for *BRCA1 *mutations were tested for *BRCA1 *rearrangements using the multiplex ligation-dependent probe amplification assay (MRC Holland, Amsterdam, The Netherlands) following the manufacturer's protocol.

### Statistical analysis

The χ^2 ^test was used to determine differences in clinicopathological features between groups. Survival curves were estimated using the Kaplan-Meier method including the log-rank test group comparison. Patients who were *BRCA1 *carriers were matched with patients with sporadic breast cancer from the Modena cancer registry. This registry, initiated in 1988, covers an area with approximately 650,000 inhabitants in Northern Italy. A database with a total of 3858 cases of sporadic breast cancer was used to find four matched controls for each case and a randomized matches were assigned between *BRCA1 *and patients with sporadic BC of the same age at diagnosis (range, between 26 and 76 ± 4 years), tumour grade (I, II, and III), and stage (I, II, and III). Multivariate analyses of DFS and OS were conducted using a proportional hazards Cox regression model. All statistical analyses were done with SPSS, version 12.0 (SPSS Inc, Chicago, IL).

## Results

### Patient Characteristics

From January 1988 and December 2006, 5923 patients were diagnosed with breast cancer. According to their family history, 4912 were considered sporadic breast cancers, 691 were at IS increased risk, and 320 were considered H risk. The patients' characteristics are detailed in Table 1. The H risk group presented with a medullary carcinoma histotype more frequently than the other two groups (*p *< .0001). The H risk group was more likely to have stage II disease than the sporadic and IS increased risk groups (*p *< .0001), and to have a significantly lower estrogen receptor (ER) and progesterone receptor (PgR) expression levels (*p *< .0001) as measured by immunohistochemical analysis (clone 6F11, Ventana, for ER; and clone 1E2, Ventana, for PgR) and stained by Ventana Benchmark autostainer. The ER and PgR receptor status was tested by evaluating the percentage of nuclear immunoreactivity with respect to all the nuclei in the neoplastic cells, independently of the staining intensity. No differences were seen in the Ki67 level between the groups (*p *= .17). The proportion of patients who received adjuvant chemotherapy was greater in the IS increased risk group than in the other two groups (*p *< .0001). The most frequent chemotherapy strategy was anthracycline-based followed by taxanes and the CMF regimen. The most frequently used first line chemotherapy regimen was platinum based, followed by a CMF scheme. The proportion of patients who received adjuvant hormone therapy was lower in the H risk group than in the other two groups, according to the negative hormonal receptor status (*p *< .0001).

**Table 1:** Clinicopathological characteristics of patients according to familial risk group

*Family Risk Group*							
	No family history		Intermediate-Slightly increased risk		High Risk		*p*
	(n = 4912)		(n = 691)		(n = 320)		
Characterics	N°	%	N°	%	N°	%	
**Histotype**							
Ductal	3936	80.1	565	81.8	251	78.4	NS
Lobular	606	12.3	83	12.0	44	13.7	NS
Tubular	93	1.9	13	1.9	2	0.6	NS
Colloid	64	1.3	10	1.4	0	0	NS
Medullary	26	0.5	6	0.9	13	4.1	< .0001
Other	187	3.9	14	2.0	10	3.2	NS

**Stage**							
I	2322	47.3	312	45.1	141	44.1	NS
II	1546	31.5	255	36.9	127	39.7	< .0001
III	802	16.4	112	16.2	48	15.0	NS
IV	216	4.4	11	1.8	1	0.3	*<*.0001
X	23	0.5	1	0.1	3	0.9	NS

**ER**							
Positive	3286	66.8	448	64.8	147	45.9	< .0001
Negative	757	15.4	139	20.2	149	46.6	< .0001
Unknown	869	17.8	103	15.0	24	7.5	< .0001

**PgR**							
Positive	2796	56.9	408	59.1	135	42.2	< .0001
Negative	1109	22.5	173	25.0	162	50.6	< .0001
Unknown	1007	20.6	110	15.9	23	7.2	< .0001

**Ki67**							
Low (0-19)	2523	51.4	383	55.4	185	57.8	NS
High (20-100) 1109		22.5	167	24.2	103	32.2	NS
Unknown	1007	20.6	141	20.4	32	10.0	NS

**Chemotherapy**							
Yes	1851	37.7	350	50.7	106	33.1	< .0001
No	3061	62.3	341	49.3	214	66.9	< .0001

**Hormone therapy**							
Yes	2804	57.1	380	55.0	106	33.1	< .0001
No	2108	42.9	311	45.0	214	66.9	< .0001

Abbreviations: ER, estrogen receptor; PgR, progesterone receptor

### Mutational Analysis

The *BRCA1 *mutational analysis was only performed in patients belonging to the H and IS risk groups, with a 22.8% (73/320) and 1.0% (7/691) mutation rate, respectively. In total, 80 patients were *BRCA1 *carriers.

### Survival analysis

**Figure 1:**
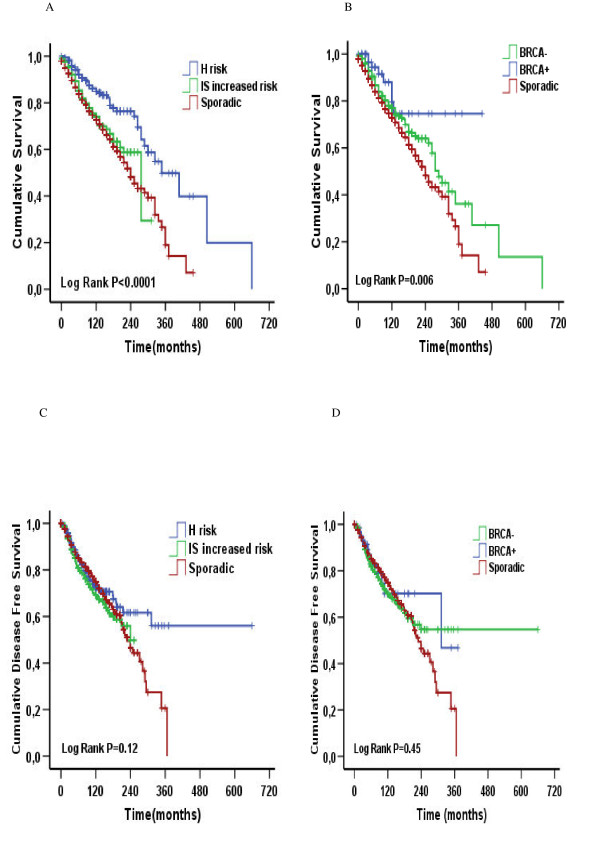
**Overall survival and disease free survival according to risk group (A, C) and to *BRCA1 *status (B, D)**.

Overall, 5923 patients were followed until December 2006, resulting in a median follow-up time of 72 months. In all, 1302 deaths (1074 sporadic, 174 IS increased risk, and 54 H risk) and 1137 relapses (884 sporadic, 186 IS increased risk, and 67 H risk) were reported. The estimated 10-year overall survival rate was 82% for the H risk patients, 75% for the IS increased risk group, and 73% for patients with sporadic breast cancer (log-rank *p *< .0001; Fig. 1A). The estimated 10-year DFS was 72% in the H risk group, 70% in the IS increased risk group, and 75% in patients with sporadic breast cancer (log-rank *p *= .12; Fig. 1C). After the genetic analysis, 80 patients were classified as *BRCA1 *carriers and 931 were considered *BRCA *negative. In the *BRCA1 *carrier group, 8 deaths (5 caused by a second tumour arising after breast cancer) and 13 relapses (6 local recurrences and 7 distant recurrences) were reported, whereas in the *BRCA *negative group 220 deaths and 240 relapses occurred. The 10-year overall survival rate was 77% for *BRCA1 *carriers, 77% for *BRCA*-negative patients, and 73% for sporadic breast cancer (log-rank *p *< .001; Fig. 1B). The 10-year DFS was 75% in sporadic breast cancer, 70% in the *BRCA*-negative patients, and 70% in the *BRCA*1 group (log-rank *p *= .45; Fig. 1D).

**Figure 2:**
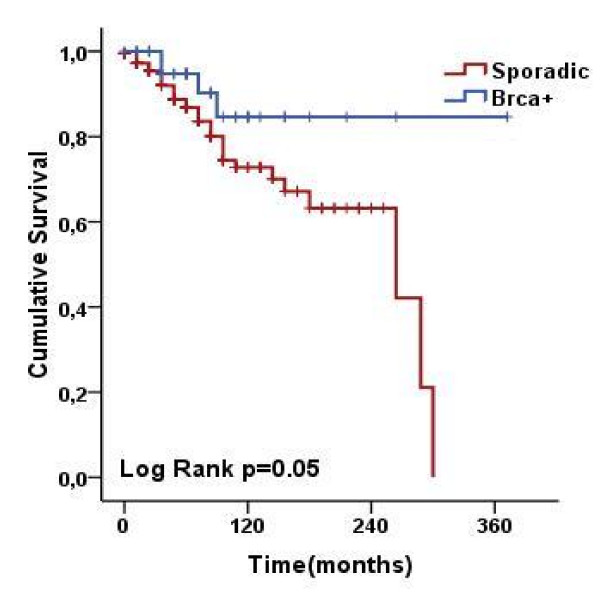
**Probability of survival in 80 *BRCA1 *breast cancer cases and 320 sporadic breast cancer controls matched for age and tumour grade and stage, using Kaplan-Meier analysis**. There was a statistically significant difference between the two groups (log-rank test, *p *= .05).

The study group of 80 *BRCA1 *cases was compared with 320 matched sporadic cases from the Modena cancer registry which were the same age, and had the same tumour grade and stage. Results are shown in Fig. 2. A statistically significant difference in OS was seen for *BRCA1 *patients compared with sporadic BC (85% vs. 73%, respectively, *p *= .05).

**Figure 3:**
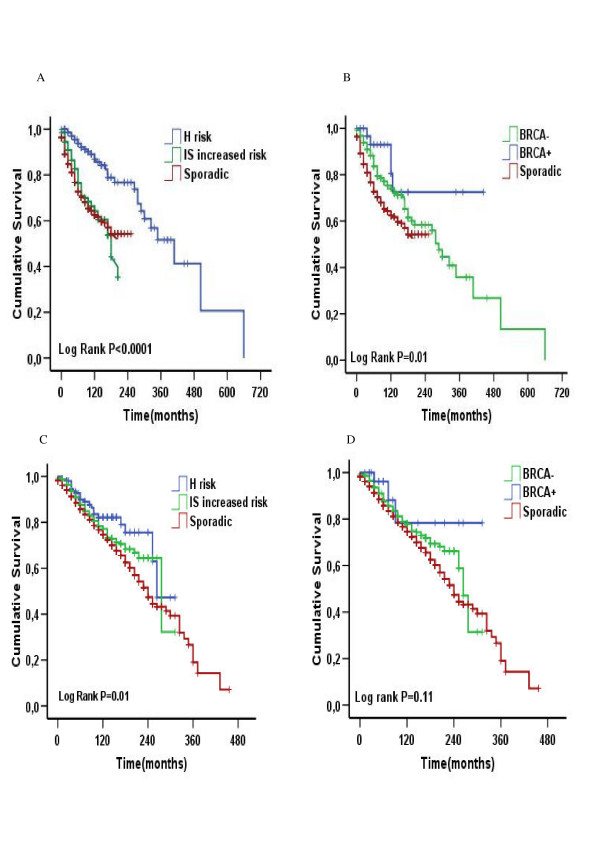
**Overall survival according to risk group in hormone receptor negative (A) and positive (C) patient subgroups, and according to *BRCA1 *status in hormone receptor negative (B) and positive (D) patient subgroups**.

According to the hormone receptor status, the hormone receptor-negative patients in the H risk group and the *BRCA1 *patients had a better OS (Fig. 3A, B) while a hormone receptor-positive advantage was only seen in the H risk group, but not in the *BRCA1 *group (Fig. 3C, D).

### Multivariate survival analysis according to prognostic factors and chemotherapy

Of the 5923 patients, 5012 were included in multivariate analyses, which included *BRCA1 *status, disease stage, ER status, PgR status, grading, age, and chemotherapy. The *BRCA1 *positive and negative status was a significant independent factor for improved OS (HR = 0.29; *p *= .002 and HR = 0.76; *p *= < .001, respectively) compared with the sporadic breast cancer cases. Positive ER and PgR status was associated with a better OS (HR = 0.82; *p *= .03 and HR = 0.79; *p *= .004, respectively). Also, chemotherapy was an important factor in reducing the mortality rate (HR = 0.68; *p *< .0001). Stage > I (HR = 3.49; *p *< .0001) and grading III (HR = 1.39; *p *< .0001) were significant factors for poorer OS. Younger age (≤ 35 years) represented a risk factor for a shorter OS, but the trend was not statistically significant. In a model that included 2213 patients receiving chemotherapy, *BRCA1*-positive status (HR = 0.38; *p *= .05) but not *BRCA*1-negative status (HR = 0.97; p = .79) reduced the mortality rate. Positive PgR status (HR = 0.76; *p *= .021) was a favourable factor as was positive ER status (HR = 0.76; *p *= .083), but did not reach statistical significance. Stage > I (HR = 3.55; *p *< .0001) and grading III (HR = 1.38; *p *< .0001) were factors statistically associated with an increased risk of death. There was a similar but non significant trend in age ≤ 35 years (HR = 1.15; *p *= .44). Conversely, in a model without chemotherapy, the *BRCA1*-positive status lost statistical significance in reducing mortality (HR = 0.23; *p *= 0.056), whereas the *BRCA1-*negative status maintained its favourable impact (HR = 0.56; *p *< .0001). Also, positive ER (HR = 0.82, *p *= .17) and PgR status (HR = 0.82; *p *= .08) and age less than 35 years (HR = 0.95;*p *= .85) did not affect OS. Finally, stage > 1 (HR = 3.43; *p *< .0001) and grading III (HR = 1.39; *p *< .0001) were statistically associated with an increased risk of death. Detailed results for multivariate analysis are shown in Table 2.

**Table 2:** Cox's proportional hazard regression models for overall survival

Variable	N° (%)	HR	*p*	[95% CI]
**All cases**	**5012 (85**)			

Sporadic	4172 (83)	1.00	-	-

BRCA -	785 (16)	0.76	< .001	0.64 to 0.89

BRCA1+	55 (1)	0.29	.002	0.13 to 0.62

stage > 1	2372 (47)	3.49	< .0001	3.01 to 4.04

Estrogen receptor positive	4090 (82)	0.82	.03	0.69 to 0.98

Progesterone receptor positive	3138 (64)	0.79	.004	0.67 to 0.93

Chemotherapy, yes	2213 (44)	0.68	< .0001	0.59 to 0.77

Age ≤ 35	199 (4)	1.12	.42	0.84 to 1.49

Grading III	1778 (35)	1.39	< .0001	1.22 to 1.58

**Model with chemotherapy**	**2213 (44)**			

Sporadic	1780 (36)	1.00	-	-

BRCA-	404 (8)	0.97	0.79	0.78 to 1.20

BRCA1+	29 (2)	0.38	.05	0.14 to 0.99

Stage > I	1689 (76)	3.55	< .0001	2.65 to 4.76

Estrogen receptor positive	1571 (71)	0.81	.083	0.65 to 1.02

Progesterone receptor positive	1492 (67)	0.76	.021	0.61 to 0.96

Age ≤ 35 years	128 (6)	1.15	.44	0.81 to 1.62

Grading III	1116 (50)	1.38	< .0001	1.15 to 1.64

**Model without chemotherapy**	**2799 (56)**			

Sporadic	2392 (85)	1.00	-	-

BRCA-	381 (14)	0.56	< .0001	0.43 to 0.73

BRCA1+	26 (1)	0.23	.056	0.14 to 1.02

stage > I	951 (34)	3.43	< .0001	2.89 to 4.09

Estrogen receptor positive	2519 (90)	0.82	.17	0.63 to 1.08

Progesterone receptor positive	2298 (82)	0.82	.08	0.65 to 1.03

Age ≤ 35	71 (3)	0.95	.85	0.56 to 1.60

Grading III	662 (24)	1.39	< .0001	1.15 to 1.68

Abbreviations: HR, hazard ratio; CI, confidence interval

**Figure 4:**
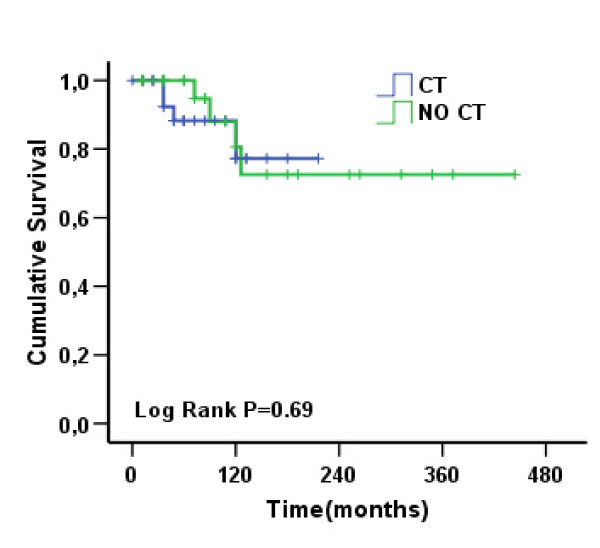
**A comparison of the group of *BRCA1 *patients who received or did not receive chemotherapy did not show any difference in overall survival (log-rank test, *p *= .69)**.

In comparing *BRCA1 *patients who did or did not receive chemotherapy, no difference was seen in terms of OS (see Fig. 4), even if all the death events in the treated group of patients were derived from second tumours (4 cases).

## Discussion

The results of this large analysis show that patients considered at H risk of being *BRCA1 *carriers had a better OS than patients considered at IS increased risk or to have sporadic breast cancer. This difference was also maintained in *BRCA1 *carriers with respect to *BRCA1*-negative and sporadic breast cancer patients. Notably, 91.2% (73/80) of *BRCA1 *carriers were identified in the H risk group, and may explain the survival advantage in this group of patients, even if other reasons may be involved. Patients who know they are at high risk of developing breast cancer may be more likely to participate in surveillance programs and start at a younger age, receive an earlier diagnosis, and subsequently, experience a better outcome.

One of the most important findings of our study was that the survival difference was attributable, in a multivariate analysis, to the patient's *BRCA1 *status and was observed in patients treated with chemotherapy. This finding is important when, as happens in a family cancer centre like in ours, we are faced with a patient or a healthy woman who has a family history of breast cancer. By collecting all the information about individuals affected by breast cancer, very long-term survivors can be identified by predicting a predisposition for being a *BRCA1-*mutation carrier in the descendants. Furthermore, *BRCA1 *carriers may be more likely to be found among women who have a high probability of hereditary breast cancer and who have a long term survival. If *BRCA1 *carriers could be identified through these characteristics, the use of expensive tests could be avoided and the rate of positive analyses increased.

Our study involves a very large number of Caucasian patients with breast cancer (N = 5923) who were first evaluated for their family history of breast cancer and subsequently for *BRCA1 *status. The most common weakness of previous studies includes the small number of patients or selection bias, such as a retrospective analysis for *BRCA1 *status. Owing to the large number of subjects, we could calculate OS differences between risk groups, and even in subgroup analyses.

Unexpectedly, the survival difference according to H risk and *BRCA1 *status was not related to the DFS. This finding shows that the H risk and *BRCA1 *patients have the same DFS as the other two groups, but seem to respond better to chemotherapeutic agents. This finding is in contrast with the results of Rennert et al. [8] who showed an insignificant difference in OS between *BRCA *carriers and non carriers and between *BRCA1 *carriers and *BRCA*-negative patients treated with chemotherapy. Our argument is that, in *BRCA1 *patients, 41% of relapses were local recurrences, whereas in patients with sporadic BC the local recurrence rate was 25%, and distant metastases accounted for 75%. Consequently, it is not surprising that a difference in OS was found and could be explained by the use of alkylating agents, such as platinum-derived drugs, in metastatic disease that are well known to be more effective in *BRCA-*related tumours [15]. Furthermore, since *BRCA1 *patients maintain a statistically significant OS advantage, even after matching each case to four controls for age and tumour grade and disease stage, this result is very important in terms of its practical value, because all confounders have been removed.

Robson et al [12] suggested that a *BRCA1 *mutation was an independent predictor of breast cancer mortality in a multivariate analysis of a group of women who did not receive chemotherapy, but not in women who received adjuvant chemotherapy.

In a multivariate analysis, we found that chemotherapy is a prognostic factor for better survival in all patients combined; furthermore, we found that a *BRCA1-*positive status was an independent predictor for a better survival in all patients and also in the subgroup of patients who received chemotherapy, but not in patients who did not receive chemotherapy. No differences are shown for *BRCA1 *patients, independently of chemotherapy or not. Also, this result might be explained by the fact that deaths in the treated group of patients were all related to a second tumour, while in the non treated group one of three deaths was caused by breast cancer. In conclusion, chemotherapy has a greater protective effect in *BRCA1 *mutation carriers compared with patients who are *BRCA*-negative and those with sporadic breast cancer. Furthermore, since *BRCA1*-related tumours are more likely to be triple negative, the greatest advantage was shown for ER-negative breast cancer, were chemotherapy is the most active. Hence, increasing the number of *BRCA1 *carriers identified by correctly selecting patients with a high probability of having hereditary breast cancer through a 10-year survival analysis could improve the benefit derived from specific chemotherapy agents (i.e., alkylating or PARP-inhibitors).

In fact, the chemotherapy benefit is well known because normal *BRCA1 *and *BRCA2 *proteins participate with *RAD51 *in the repair of double-stranded DNA breaks induced by DNA-damaging agents [16-19]. The better prognosis could be due to the deficiency of the *BRCA *proteins, which confer substantial cellular sensitivity to the inhibition of poly (ADP-Ribose) polymerase enzyme (PARP). This polymerase is a key enzyme in the repair of single-stranded DNA damage via the base excision repair pathway. The loss of PARP activity in *BRCA *mutant cells might lead to the persistence of DNA lesions normally repaired by homologous recombination, resulting in increased chromosome instability and programmed cell death specifically in tumour cells [20,21]. Therefore, because there is no functional protein within the tumour cells, they lose their capacity to repair DNA damage. This might be specifically pronounced for drugs, such as cisplatin, acting through the induction of DNA damage leading to cell death and to a better therapeutic response. In a small clinical study, Chappuis et al [22] found a benefit in Ashkenazi Jews with locally advanced breast cancer who were *BRCA*-positive and treated with anthracycline-based chemotherapy regimens. In this study, all patients received anthracycline-based neoadjuvant chemotherapy, and 10 of 11 patients with *BRCA *mutations had a clinical complete response compared with only 8 of 27 *BRCA*-negative patients with sporadic breast tumours. From this study, it was inferred that tumours with *BRCA1 *mutations are highly sensitive to anthracycline-based chemotherapy regimens.

In our study, patients were considered part of a hospital-based population and were not selected for age, tumour stage, or treatment, which could influence survival rates. We were successful in obtaining blood samples from all patients and were able to genotype all the samples we received. The patients were followed for a median of 72 months. Treatment regimens were chosen on the basis of disease staging. The Modena Cancer Registry captures outcome data from almost all patients with cancer who are treated in the province. Data on pathological diagnosis are verified by a pathologist, and incomplete information is retrieved from medical records when possible. All patients were Caucasian, and it is possible that the chemotherapy sensitivity can be associated with specific modifier genes not found in other populations.

Our study has a number of limitations. Tumour stage and hormone receptor status were not routinely recorded, particularly during the first period. Since we tested only a subpopulation of patients with a positive family history of breast cancer, it is possible that some hereditary cases were misclassified; but we found that only 1% of mutations occurred in the IS increased risk group suggesting that the number of *BRCA1 *carriers in the sporadic group would be very low. Since we identified only 80 mutation carriers, the subgroup analysis relied on a small number of subjects.

In conclusion, our study is the first to evaluate a relationship between familial risk of breast cancer and a genetic assessment in terms of DFS and OS. We propose that the increased survival associated with a family history of breast cancer, suggesting hereditary breast cancer according to the Modena criteria, should be considered with respect to *BRCA1 *analysis as a predictor of mutations. We feel that randomized studies need to be designed to further address the relationship between *BRCA1 *mutations and sensitivity to chemotherapy, and further clinical investigations are required to determine whether the *BRCA1 *status can be used to predict treatment outcomes.

## Conclusions

In conclusion, our data show that patients belonging to a population with a high probability of being *BRCA1 *carriers have a better prognosis than other risk groups, and this could be considered an indicator of *BRCA1 *inheritance. This paper provides, for the first time, evidence-based proof that women at high risk of being *BRCA1 *carriers have a favorable prognosis after 10 years follow-up.

## Competing interests

The authors declare that they have no competing interests.

## Authors' contributions

LC participated in the design of the study and drafted the manuscript. CM acquired the data and performed the statistical analysis. CC participated in the statistical analysis. VM performed the genetic testing. IM acquired the data from the centre. GC participated in the collection of patient data from Mantua Hospital. GP participated in the collection of patient data from Rimini Hospital.

DT participated in the collection of patient data from Bologna Hospital. MF designed the study and revised the final manuscript. All authors read and approved the final manuscript.

## Pre-publication history

The pre-publication history for this paper can be accessed here:

http://www.biomedcentral.com/1471-2407/10/90/prepub
